# Bridgedd, a Health Care Staff–Facing Community Resource Navigation Tool for an Urban Safety-Net Hospital: Cross-Sectional Formative Pilot Study

**DOI:** 10.2196/101610

**Published:** 2026-10-19

**Authors:** Aravind Venkatachalam, Charles E Moore

**Affiliations:** 1 Department of Otolaryngology, Grady Health System, Emory University, 49 Jesse Hill Jr DriveAtlanta, GA, 30303, United States, 1 4047783801

**Keywords:** community resources, social determinants of health, mental health, safety-net hospital, digital health, formative pilot, quality improvement

## Abstract

**Background:**

Health care professionals in safety-net settings often rely on lengthy and inconsistently maintained community health resource lists when connecting patients with services. Bridgedd is a health care staff–facing web application designed to generate geographically and categorically filtered community resources in a unique format that makes information about each resource more accessible.

**Objective:**

This formative pilot study aimed to characterize the perceptions of behavioral health professionals toward Bridgedd’s usability, comparative value relative to existing community resource filtering and communication tools, and their intent to use it with patients if made available.

**Methods:**

We conducted a single-site, cross-sectional formative pilot of the web-based tool we developed, Bridgedd, within Grady Health System’s Integrated Behavioral Health Program between April 23 and May 14, 2026. All 21 program team members attending an all-staff meeting received a presentation about Bridgedd and were given the opportunity to try the tool and ask questions. Then, they independently evaluated it using a self-generated hypothetical patient scenario. Eleven completed an investigator-developed anonymous feedback survey assessing perceived ease of use, comparative value relative to existing tools, intent to use the tool with patients, and open-ended feedback. Quantitative responses were summarized using raw counts, and open-ended responses were summarized as descriptive observations. A post hoc descriptive analysis excluded 2 potentially direction-confused response patterns. The post hoc descriptive analysis did not materially change the overall interpretation.

**Results:**

Eleven behavioral health professionals (therapists, n=4; licensed professional counselors, n=3; social workers, n=2; mental health counselor, n=1; certified peer specialist–mental health, n=1) completed the survey. Of the 11 respondents, 9 rated the tool as very easy to use, none reported technical problems, 8 agreed or strongly agreed that Bridgedd was better than the tools they currently use to help patients navigate community health resources, and 8 reported an intent to use the tool with patients if it were made available. In the open-ended responses, 7 of the 11 respondents suggested that nothing was missing or offered no additional suggestions for improvement, while the remaining 4 open-ended responses centered on expanding mental or behavioral health resources, providing additional county coverage, including more resources within each category, and building a mechanism for updating resource information.

**Conclusions:**

In this small, single-setting formative pilot study using hypothetical patient scenarios, participating behavioral health professionals generally reported favorable perceptions of Bridgedd’s usability and potential utility. Further evaluation during routine clinical care is needed to assess actual use, workflow effects, patient experience, referral completion, and patient-level outcomes.

## Introduction

Patients receiving care in safety-net settings may encounter barriers when trying to take advantage of community resources such as food assistance, transportation services, free clinics, housing, and behavioral health services. Although health care systems have increasingly implemented processes for identifying health-related social needs, the process of translating those identified needs into successful connections with community services remains a challenge [1-3]. Even when referrals are offered, patients may decline services or be lost to follow-up, indicating that identifying a resource does not necessarily result in a successful connection to that resource [4]. To address this challenge, technology-enabled closed-loop referral initiatives have been developed to help coordinate health care and social service connections [5].

One example is the use of electronic community resource referral systems. These systems have become more common, and reviews of them highlight workflow integration, maintenance of current community resource inventories, and partnerships with community-based organizations as important implementation considerations. These reviews also identify technical challenges and costs as barriers to adopting electronic systems [6-8].

Additionally, patient-facing materials could introduce a new access barrier. In an analysis of English-language Epic discharge documents, 81% exceeded the American Medical Association’s recommended sixth-grade reading level on the Flesch-Kincaid grade level scale, and 100% exceeded it on the Simple Measure of Gobbledygook [9]. This means that patient-facing written materials might act as an additional barrier when they are difficult to understand or act upon. Additionally, there have been studies that also have identified variation in the quality and equity of discharge instructions [10].

One area of health care in which patients are often connected to community health resources is behavioral health. Behavioral health professionals may need to connect patients with resources that address immediate psychological needs [11]. It has also been found that access to office-based specialty mental health resources varies across communities and is lower in low-income areas [12]. This presents the need for a tool that helps health care staff identify and communicate locally relevant resources to patients in an actionable format.

To address this gap, we developed Bridgedd, a health care staff–facing web application that allows clinicians to generate filtered, printed community resource flyers organized by county and category of resources. Our priority was making sure that the tool was easily accessible, presented information relevant to common barriers such as transportation, cost, and unclear next steps in a visual and easy-to-understand format, and generated a printable output that health care staff could quickly hand to a patient [1-3].

The objective of this single-site formative pilot study was to characterize behavioral health professionals’ perceptions of Bridgedd’s usability, comparative value relative to existing community resource filtering and subsequent patient communication tools, and intent to use it with patients if made available.

## Methods

### Study Design and Setting

We conducted a single-site, cross-sectional formative pilot study of Bridgedd at Grady Memorial Hospital in Atlanta, Georgia, between April 23 and May 14, 2026. Grady is one of the largest urban safety-net hospitals in the United States and serves a patient population with high rates of lack of insurance, limited English proficiency, and unmet social needs. The tool was presented at a behavioral health clinic team meeting with 21 members, 11 of whom completed the survey. The behavioral health clinic team was established in 2018, and it initially served Grady’s primary care centers and neighborhood health centers and subsequently expanded to specialty settings, including the cancer, burn, sickle cell, obstetrics-gynecology, liver, diabetes, and gender clinics. At the time of evaluation, the program roster had 21 team members, with 17 licensed therapists and 4 certified peer specialists. Participants evaluated Bridgedd independently using self-generated hypothetical patient scenarios. This approach allowed participants to conduct a preliminary evaluation of the tool without disrupting clinical care and to inform future testing.

### The Bridgedd Tool

Bridgedd is a web-based application that allows health care staff to generate filtered, printable community resource flyers at the point of care. Health care staff select a county within metro Atlanta (Fulton, DeKalb, Clayton, Gwinnett, or Cobb) and a resource category (federally qualified health centers, food assistance, free clinics, benefits navigation, health departments, or diabetes education).

The tool then returns a curated, formatted flyer listing local resources with the organization name, address, phone number, hours, eligibility criteria, languages served, and cost. Each resource entry is accompanied by toggleable supplemental information addressing common barriers faced in accessing these resources such as transportation, cost, and unclear next steps (Figure 1).

**Figure 1.**
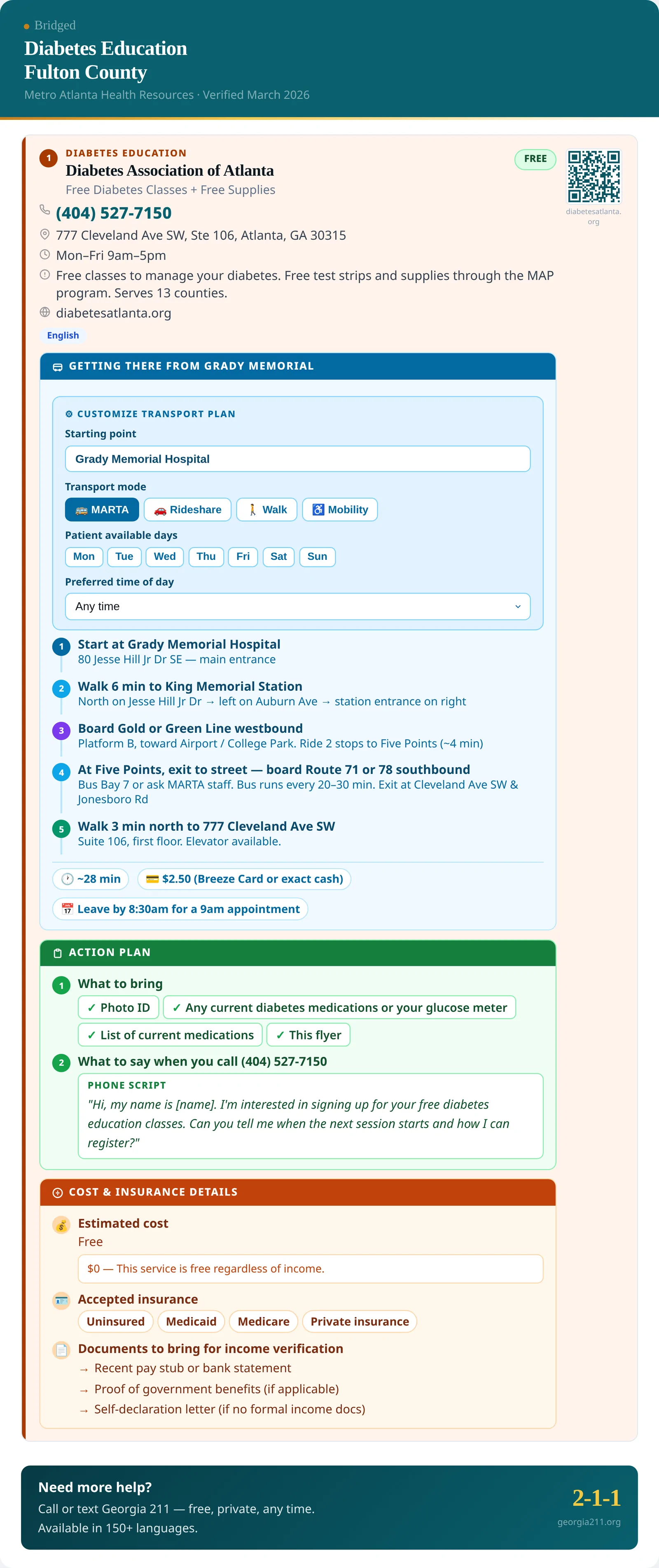
Example of the Bridgedd health care staff interface and generated community resource flyer evaluated by behavioral health professionals during a single-site formative pilot study at Grady Memorial Hospital in Atlanta, Georgia, April 23 to May 14, 2026.

The tool runs in a standard web browser, requires no login, and generates a print-ready output that health care staff can hand to patients during clinical encounters. The tool is also compatible with mobile devices. At the time of the pilot study, the Bridgedd database contained 33 manually curated community resources across the previously mentioned 6 categories and 5 metro counties. Each resource entry contained 17 structured fields, including organization name, address, phone number, hours, cost structure, accepted insurance and income documentation, languages served, and a “last verified” date. Resource entries are maintained in a structured database with a “last verified” field, and at the time of the pilot study, the dataset reflected information verified in March 2026. Bridgedd is designed to be used by health care staff during clinical encounters, but during this pilot study, live encounters with patients were not evaluated.

### Participants and Recruitment

The 21 integrated behavioral health program team members attended a scheduled all-staff meeting at which Bridgedd was presented. Meeting attendees included the previously mentioned licensed therapists and certified peer specialists working within Grady’s primary care, neighborhood health, specialty clinics, or employee-support settings. All 21 attendees were invited to participate in the evaluation of the tool.

### Procedure

During the all-staff meeting, the study team introduced Bridgedd and demonstrated its primary functions. Following the presentation, all 21 attendees were given access to the tool and were shown how to use it. Then, they were asked to use the tool to generate at least one resource flyer relevant to a typical patient encounter. They independently generated a scenario of their own choice without observation. After interacting with the tool, participants completed a brief structured feedback survey administered via a web form. Whether participants completed the survey immediately after using the tool or sometime afterward was not measured. The survey was open between April 23 and May 14, 2026.

The survey was hosted as a single-page Google Form and was open from April 23 to May 14, 2026. All quantitative items were configured as required fields, and the open-ended item was optional. Respondents could review and modify their answers before submission. No login was required, and no technical safeguards, such as IP tracking or cookies, were used to prevent duplicate submissions. Responses were stored in a linked Google Sheet accessible only to the study team. Also, participation was voluntary and uncompensated.

### Measures

This pilot study used an investigator-developed survey that had 6 items: (1) self-reported health care staff role, (2) perceived ease of use on a 5-point scale (1=“very easy,” 5=“very difficult”), (3) presence of technical problems (free text), (4) agreement that “this tool is better than the current tools I use to help patients navigate community health resources” on a 5-point Likert scale (1=“strongly disagree,” 5=“strongly agree”), (5) intent to use the tool with patients if made available, on the same 5-point scale, and (6) an open-ended item to capture what was missing from the tool and what improvements would be desired.

The survey instrument was specifically developed for this evaluation and was not pilot-tested or validated before administration. Additionally, the 3 quantitative measures were separate formative feedback measures rather than part of a larger composite scale including all 3 measures. The full survey instrument is provided in Multimedia Appendix 1.

### Data Analysis

The quantitative survey responses were summarized descriptively using raw response counts. Because the sample size was very small, no inferential statistical testing was performed. The 3 quantitative items were required in the survey, so each item had 11 responses. The open-ended item was optional; however, all participants responded to it, and responses were summarized.

The ease-of-use item was oriented so that lower values represented greater perceived ease, whereas higher values represented more favorable responses for the comparative value and intent to use items. The ease-of-use item was reverse coded so that higher values represented more favorable responses across all 3 items.

The full sample of 11 respondents was used in the primary descriptive analysis. Two of the respondents selected the lowest response value for all 3 measures but then provided nonnegative open-ended responses. Since their intended responses could not be verified and there was no prespecified exclusion criterion for this pattern, we did not remove these responses from the primary analysis. However, we conducted a post hoc descriptive analysis excluding these responses.

The post hoc descriptive analysis included the remaining 9 respondents and was used to understand whether the potentially confused patterns affected the overall interpretation. However, since this exclusion was performed after looking at the responses, the post hoc descriptive analysis was of a more exploratory nature.

The open-ended responses were reviewed and summarized descriptively. The responses were grouped according to the specific suggestions or observations reported, and the number of responses that mentioned each suggestion was recorded. No formal thematic analysis was conducted. This study was reported in accordance with CHERRIES (Checklist for Reporting Results of Internet E-Surveys; Checklist 1).

### Ethical Considerations

This project was determined to be nonhuman subjects research by the Emory University Institutional Review Board (IRB) on the basis that the project did not meet the federal definition of research designed to develop or contribute to generalizable knowledge (45 CFR 46.102(l)). The project was classified as a program evaluation. No IRB submission or approval was therefore required, and Emory’s nonhuman subjects research determination form is on file with the project leader. Participating health care staff were informed that the survey was voluntary, and survey completion was treated as implied consent. No patient-level data were collected, and health care staff responses were anonymous.

## Results

### Sample

Eleven behavioral health professionals at Grady Memorial Hospital completed the feedback survey between April 23 and May 14, 2026, out of 21 eligible attendees at the behavioral health clinic team meeting. Respondents included therapists (n=4), licensed professional counselors (n=3), social workers (n=2), a mental health counselor (n=1), and a certified peer specialist–mental health (n=1).

### Usability

Of 11 respondents, 9 rated the tool as “very easy” to use, the most favorable response option. One respondent rated the tool as “very difficult” to use, and 1 respondent rated the tool’s ease of use as neutral. No respondents reported experiencing a technical problem while using the tool. The complete response distribution is shown in Table 1.

**Table 1.** Distribution of health care staff responses to 3 quantitative measures following independent use of Bridgedd with hypothetical patient scenarios during a single-site, cross-sectional formative pilot study at Grady Memorial Hospital in Atlanta, Georgia, April 23 to May 14, 2026 (N=11).

Survey item	Very unfavorable, n	Unfavorable, n	Neutral, n	Favorable, n	Very favorable, n
Perceived ease of use	1	0	1	0	9
Comparative value	2	0	1	2	6
Intent to use	2	0	1	0	8

### Comparison to Existing Tools

Of 11 respondents, 8 agreed or strongly agreed that Bridgedd was better than their current tools. Of these 8 respondents, 6 strongly agreed and 2 agreed. One respondent selected the neutral point, and 2 strongly disagreed.

### Intent to Use

Of 11 respondents, 8 reported that they would use Bridgedd with patients if it were made available. One respondent chose the neutral midpoint, and 2 strongly disagreed.

### Open-Ended Feedback

All 11 respondents answered the optional question, and 7 indicated that there was nothing missing from the tool or offered no additional suggestions. First, health care staff requested expanded geographic coverage, including resources in additional counties beyond the current metro Atlanta scope. Specifically, 2 respondents requested expanded mental or behavioral health resources. One of these respondents specifically identified Claratel Behavioral Health & Addiction & Recovery and Mercy Care as examples of desired organizations.

Second, health care staff asked for greater depth within each category. One respondent requested at least 5 options within each resource category and a dedicated list of mental health clinics by county. Finally, 1 respondent suggested a mechanism for updating the resource information. Because these responses were open-ended and each respondent could make multiple suggestions, the counts are not mutually exclusive.

### Post Hoc Descriptive Analysis

In the post hoc descriptive analysis, the 2 potentially direction-confused response patterns were excluded. The analysis showed that 7 of 9 respondents rated Bridgedd as very easy to use, 1 selected the neutral midpoint, and 1 rated it as very difficult. In total, 8 of 9 agreed or strongly agreed that Bridgedd was better than their current tools, and 8 of 9 strongly agreed that they would use Bridgedd with patients if it were made available. One respondent selected the neutral midpoint for both the comparative value and intent-to-use measures. Excluding these 2 response patterns did not materially change the overall interpretation. The perceived ease of use remained favorable, while the proportions reporting favorable comparative value and intent to use increased.

## Discussion

### Principal Findings

The objective of this formative pilot study was to characterize the perceptions of behavioral health professionals of Bridgedd’s usability, comparative value relative to existing community resource filtering and communication tools, and their intent to use it with patients if made available.

The 11 respondents generally reported favorable perceptions across these 3 measures. Nine rated Bridgedd as very easy to use, 8 rated it as favorable relative to their existing tools, and 8 indicated that they would use it with patients if it were made available. None of the respondents reported technical problems, and a post hoc descriptive analysis excluding 2 potentially direction-confused response patterns did not materially change the overall interpretation.

Although these results are encouraging, it is important to note that health care staff used hypothetical scenarios and did not use Bridgedd during clinical encounters. Thus, these results serve as preliminary findings from a formative pilot study aimed at informing future pilot studies.

### Comparison With Prior Work and Implications

These findings are consistent with prior research on electronic community resource referral systems, which has identified workflow integration, technical fit, resource maintenance, and organizational context as important implementation considerations [4-6]. However, as stated previously, because the tool was not used during live patient encounters, the stated intent to use is not equivalent to adoption and sustained use. Subsequent evaluation during routine clinical use of the tool is necessary to determine whether there is a discrepancy between intent to use and actual use.

### Open-Ended Feedback

The open-ended responses provided preliminary descriptive observations that could inform future improvements. They identified resource breadth, geographic coverage, and information maintenance as important considerations before implementation. There were requests to add additional counties and provide greater depth within the resource categories, which suggests that the tool’s usefulness might depend on whether the database reflects the geographic and clinical scope of health care staff’s work. Also, there was a request to update the resource information, which highlights the need for a sustainable process to maintain accuracy as information changes over time.

### Limitations

This pilot study has several limitations. The respondent group was small and from a single program within one health system. Next, although all 21 program team members attended the presentation and were invited to participate, we were only able to get responses from 11 participants. Also, because the survey was anonymous, we were unable to systematically compare respondents with nonrespondents, so those who responded might have simply had more positive views of Bridgedd. As a result, the results should not be assumed to represent all invited team members or professionals in other settings.

Additionally, the survey used was investigator-developed, and it was not pilot-tested or validated before administration. The mixed-direction response scale could have contributed to the response-direction confusion. Additionally, the post hoc descriptive analysis done by excluding the 2 potentially direction-confused responses was developed after the responses were already received and reviewed. As previously stated, the intended responses of these 2 respondents could not be verified. Thus, the post hoc descriptive analysis should be considered exploratory.

Another limitation lies in the study setting. The respondents used Bridgedd independently with hypothetical scenarios rather than during live clinical encounters, and consequently, the evaluation did not reproduce clinical time pressure, interruptions, other responsibilities, urgent patient needs, or other workflow conditions. Also, the tool use was not observed, nor was the time interval between tool use and survey completion measured. Thus, the findings indicate self-reported perceptions and intent.

### Future Directions

Further evaluations should include direct patient feedback regarding whether the features included, such as transportation information, action plans, and cost details, are actually viewed by patients as increasing accessibility. The next steps for Bridgedd include adding more resources to the database and piloting it with a wider range of health care staff and settings, as well as during real clinical encounters. Future studies should collect objective measures such as tool-use frequency, time required to generate a flyer, and workflow effects. Additionally, adding a patient-facing evaluation to assess accessibility, perceived usefulness, and resource use would be warranted.

### Conclusions

In this formative pilot study at a large urban safety-net hospital, behavioral health professionals generally reported favorable perceptions of Bridgedd’s usability and potential utility. These preliminary findings support continued iterative development and planned expansion to broader health care staff populations and patient-level outcome measurement. If future studies show similar results in routine clinical care, this could indicate that a health care staff–facing tool that generates locally tailored, printable resources could help safety-net health care staff communicate information about community resource options more efficiently. Future work should focus on determining how to integrate Bridgedd into existing clinical workflows and improve patient access, referral completion, and resource use.

## Supplementary material

10.2196/101610 Multimedia Appendix 1 Survey instrument developed by study team.

10.2196/101610 Checklist 1 CHERRIES checklist.

## Abbreviations

CHERRIES: Checklist for Reporting Results of Internet E-Surveys
IRB: institutional review board
