# Supplementary material for: Bridgedd, a Health Care Staff–Facing Community Resource Navigation Tool for an Urban Safety-Net Hospital: Cross-Sectional Formative Pilot Study
Source: JMIR Form Res. 2026 Oct 19;10:e101610. doi: 10.2196/101610 (PMC13679872; doi:10.2196/101610)
Supplement: Supplementary file 1 [file formative-v10-e101610-s001.pdf]

Multimedia Appendix 2

Bridgedd Provider Feedback Survey Instrument

Manuscript: Bridgedd: A Provider-Facing Community Resource Navigation Tool for an Urban Safety-Net Hospital: A Feasibility and Acceptability Pilot Study

Platform: Google Forms (single-page web form)

Administration period: April 23, 2026 – May 14, 2026

Number of items: 6 (3 closed-ended scaled items, 3 free-text items)

Estimated completion time: 2–3 minutes

Survey introduction

Thank you for taking a few minutes to share your feedback on Bridgedd, a tool designed to help providers generate community resource flyers for patients. Your responses are anonymous and will be used to guide further development of the tool. Participation is voluntary. Completing this survey indicates your consent to participate.

Item 1. Provider role

Question: What is your role?

Response format: Short-answer text (free-text)

Required: Yes

Item 2. Ease of use

Question: How easy was the tool to use?

Response format: 5-point linear scale

**1**	**2**	**3**	**4**	**5**	
Very easy				Very difficult	

Required: Yes

Note: For this item, lower scores indicate better usability. This scale direction is opposite to that of Items 4 and 5.

Item 3. Technical problems

Question: Did you encounter any technical problems?

Response format: Short-answer text (free-text)

Required: Yes

Item 4. Comparative value

Question: This tool is better than the current tools I use to help patients navigate community health resources.

Response format: 5-point Likert agreement scale

1	2	3	4	5	
Strongly disagree				Strongly agree	

Required: Yes

Item 5. Adoption intent

Question: I would use this tool with patients if made available.

Response format: 5-point Likert agreement scale

1	2	3	4	5	
Strongly disagree				Strongly agree	

Required: Yes

Item 6. Open-ended feedback

Question: What is missing? What else would you like to see in this tool?

Response format: Long-answer text (free-text)

Required: No (optional)

Administration notes

The survey was hosted as a single-page Google Form and was accessed via a link distributed at an in-person Behavioral Health clinic team meeting at Grady Memorial Hospital. All items were presented on a single screen that they could scroll down on. Respondents could review and modify their answers before submission, consistent with the default behavior of Google Forms. No respondent identifiers were collected, and no cookies or IP-based tracking were used. The survey was not pilot tested prior to administration. Items 2 (ease of use) and Items 4 and 5 (Likert agreement) used opposing scale directions, with lower scores reflecting better usability for Item 2 and higher scores reflecting greater agreement for Items 4 and 5. This mixed-direction scaling is acknowledged in the manuscript. The limitations section has this as a potential source of measurement error.
