# Supplementary material for: Bridgedd, a Health Care Staff–Facing Community Resource Navigation Tool for an Urban Safety-Net Hospital: Cross-Sectional Formative Pilot Study
Source: JMIR Form Res. 2026 Oct 19;10:e101610. doi: 10.2196/101610 (PMC13679872; doi:10.2196/101610)
Supplement: Supplementary file 2 [file formative-v10-e101610-s002.pdf]

Multimedia Appendix 1

CHERRIES Checklist for Reporting Results of Internet E-Surveys

This study was reported in accordance with the Checklist for Reporting Results of Internet E-Surveys (CHERRIES), as proposed by Eysenbach (2004).

Reference: Eysenbach G. Improving the Quality of Web Surveys: the Checklist for Reporting Results of Internet E-Surveys (CHERRIES). J Med Internet Res. 2004;6(3):e34. doi:10.2196/jmir.6.3.e34

Manuscript: Bridgedd: A Provider-Facing Community Resource Navigation Tool for an Urban Safety-Net Hospital: A Feasibility and Acceptability Pilot Study

Item	Category / Question	Response
Design		
1	Describe survey design	Cross-sectional, single-site, provider-facing feedback survey administered after participants used the Bridgedd web application. Investigator-developed instrument; not previously validated. Reported in Methods (Study design and setting; Measures).
IRB approval and informed consent		
2	IRB approval	This project was determined to be non-human subjects research by the Emory University Institutional Review Board, on the basis that it did not meet the federal definition of research designed to develop or contribute to generalizable knowledge (45 CFR 46.102(l)). The project was classified as a program evaluation. No IRB submission or approval was required. The Emory non-human subjects research determination form is on file with the project leader. Reported in Methods (Ethical considerations).
3	Informed consent	Participating providers were informed that the survey was voluntary, and survey completion was treated as implied consent. No identifying patient data were collected, and provider responses were anonymous. Reported in Methods (Ethical considerations; Participants).
4	Data protection	Survey responses were collected via a Google Forms web form and stored in a linked Google Sheet accessible only to the study team. No provider identifiers were collected, and no patient-level data were collected.
Development and pre-testing		

Item	Category / Question	Response
5	Development and testing	The survey instrument was investigator-developed and was not pilot tested prior to administration. This is explicitly disclosed in Methods (Measures) and acknowledged in the Limitations. The full instrument is provided in Multimedia Appendix 2.
Recruitment process and description of the sample		
6	Open survey vs closed survey	Closed survey. Access was limited to attendees of a single Behavioral Health clinic team meeting at Grady Memorial Hospital (23 eligible attendees).
7	Contact mode	Participants were recruited in person at a Behavioral Health clinic team meeting. The Bridgedd tool was presented at the meeting, and providers were given access to the tool and the feedback survey link.
8	Advertising the survey	The survey was not publicly advertised. Recruitment was confined to the in-person team meeting.
Survey administration		
9	Web/email	Web-based survey administered via Google Forms.
10	Context	The survey was hosted as a single-page Google Form, accessed via a link shared at the team meeting.
11	Mandatory/voluntary	Participation was voluntary and uncompensated. Reported in Methods (Participants).
12	Incentives	None. No compensation was offered. Reported in Methods (Participants).
13	Time/date	The survey was open from April 23, 2026 through May 14, 2026. Reported in Methods (Procedure) and Results (Sample).
14	Randomization of items or questionnaires	Not applicable. The survey contained six fixed items presented in a single order.
15	Adaptive questioning	Not used. All participants saw all six items.
16	Number of items	Six items: (1) provider role, (2) ease of use, (3) technical problems (free-text), (4) agreement that Bridgedd is better than current tools, (5) intent to use the tool with patients, and (6) open-ended feedback on missing features. The full instrument is provided in Multimedia Appendix 2.
17	Number of screens (pages)	One screen. All six items were presented on a single page.

Item	Category / Question	Response
18	Completeness check	All quantitative items were configured as required fields. The open-ended free-text item was optional. No partial responses were recorded.
19	Review step	Respondents could review and modify their answers before submission, consistent with the default behavior of Google Forms.
Response rates		
20	Unique site visitor	Not tracked separately. The survey link was distributed only to the 23 attendees of the team meeting.
21	View rate	Not measured. The absence of objective usage metrics is acknowledged in the Limitations.
22	Participation rate	Not separately measured. Survey access and survey completion were treated as a single step.
23	Completion rate	All 11 respondents who began the survey completed it (100% completion rate among submitters). Overall response rate from the eligible population was 11 of 23 (48%).
Preventing multiple entries from the same individual		
24	Cookies used	No cookies were used to identify or track respondents. The closed, in-person recruitment context made duplicate submissions unlikely.
25	IP check	IP addresses were not checked. The closed, in-person recruitment context made duplicate submissions unlikely.
26	Log file analysis	Not conducted. No objective usage or timing metrics were collected from the survey platform. Acknowledged in the Limitations.
27	Registration	Not used. The survey did not require login or registration.
Analysis		
28	Handling of incomplete questionnaires	Not applicable. No incomplete questionnaires were submitted. All 11 respondents completed all quantitative items.
29	Questionnaires submitted with an atypical timestamp	Not analyzed separately. Two responses showed a pattern consistent with scale-direction confusion (uniform '1' ratings paired with neutral or positive open-ended feedback). This is reported in Results (Response patterns) and discussed in Limitations. No respondents were excluded from analysis.
30	Statistical correction	No imputation or statistical correction was performed. Given the pilot nature and small sample size (N=11), only descriptive

Item	Category / Question	Response
		statistics (mean, median, SD, IQR, and frequency distribution) were calculated, and no inferential testing was conducted. Reported in Methods (Analysis).

Note: Items marked "not applicable" or "not measured" reflect the closed, in-person, pilot nature of the study, in which the survey was distributed only to a known group of meeting attendees. The absence of automated tracking and the small sample size are discussed in the manuscript Limitations section.
